# Twenty years' use of expanded polytetrafluoroethylene sheet for an artificial cardiac pacemaker

**DOI:** 10.1002/ccr3.4334

**Published:** 2021-06-24

**Authors:** Kenji Yorita, Junichi Takahashi, Kazutoshi Tano, Yoichi Ichikawa, Nobumasa Hamaguchi, Wataru Yasui

**Affiliations:** ^1^ Department of Diagnostic Pathology Japanese Red Cross Kochi Hospital Kochi‐city Japan; ^2^ Department of Internal Medicine Japanese Red Cross Kochi Hospital Kochi‐city Japan; ^3^ Department of Cardiovascular Surgery Japanese Red Cross Kochi Hospital Kochi‐city Japan; ^4^ Department of Thoracic Surgery Japanese Red Cross Kochi Hospital Kochi‐city Japan; ^5^ Department of Internal Medicine Yusuhara Hospital Kochi‐city Japan

**Keywords:** cardiac pacemaker, expanded polytetrafluoroethylene sheet, foreign body

## Abstract

Physicians can prolongedly use expanded polytetrafluoroethylene sheets for fixation of artificial cardiac pacemakers to avoid pacemaker lead displacement. The sheets can also be used to prevent implant rejection in patients with metal allergies.

## Graphical abstract

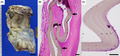

## 1 WHAT MATERIAL ENCASES AN ARTIFICIAL CARDIAC PACEMAKER?

**FIGURE 1:**
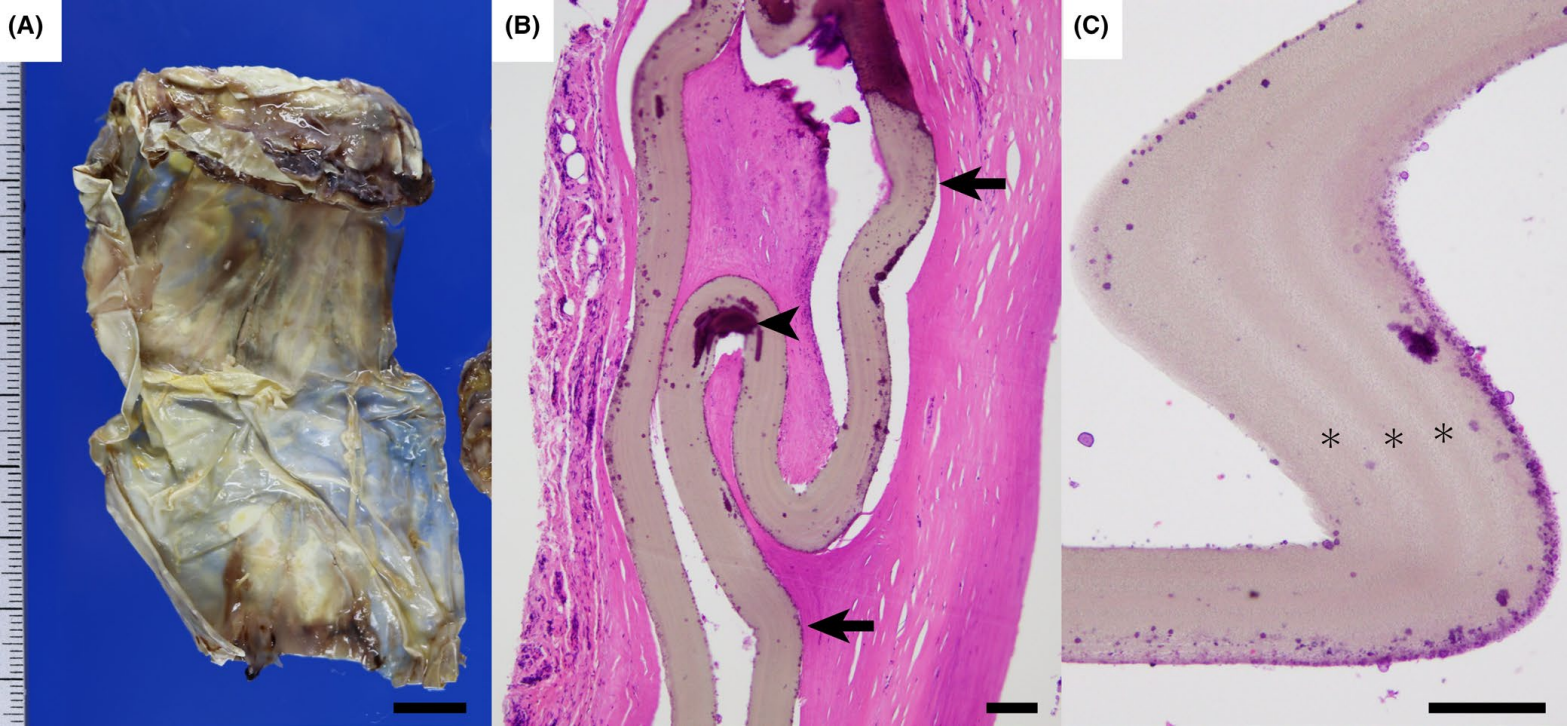
The membranous material, which formerly encased the cardiac pacemaker (A). Histologically, a foreign body with a thickness of 0.1 mm is embedded in a dense fibrous stroma (B). The material and calcification are indicated by arrows and arrowheads, respectively. The higher magnification image of the material shows a laminar structure indicated by asterisks. Scale bar in A, B, and C is 1 cm, 0.1 mm, and 0.1 mm, respectively

An 84‐year‐old Japanese woman was admitted to our hospital to have her artificial cardiac pacemaker's battery replaced. The pacemaker had been inserted for sick sinus syndrome 20 years ago, and the battery had been replaced 11 years ago. Shortly after the insertion of the pacemaker, the patient had a history of four surgeries for the adjustment of the pacemaker lead position. During the second battery replacement, a physician noticed a hard, membranous material encasing the pacemaker. This material was removed (Figure 1A) to reduce the risk of infection. Histological analysis revealed a foreign body, which resembled an expanded polytetrafluoroethylene (ePTFE) sheet with a 0.1 mm‐thick laminar structure, embedded in a fibrous stroma (Figure 1B–1C).^1^ We were unable to obtain data concerning the exact time of insertion of the ePTFE sheet due to the lack of medical records. It must have been inserted during one of the previous four surgeries and had probably been used for 20 years. The purpose of its use was unknown; however, the ePTFE sheet was likely used for fixation of the pacemaker to avoid pacemaker lead displacement. The sheets also can be used to prevent implant rejection in patients with metal allergies.^2^ The longstanding use of the sheet was likely related to lack of adverse events of the inserted material and physician's unawareness due to the loss of medical records. Our case likely demonstrates the safety of prolonged use of ePTFE sheets with artificial pacemakers.

## CONFLICT OF INTEREST

The authors state that they have no conflicts of interest to declare or funding resources to mention.

## AUTHOR CONTRIBUTIONS

KY: drafted the manuscript and contributed to the pathological diagnosis of the foreign body. JT: treated the patient and removed the foreign body. KT, YI, and NH: assisted with the analysis and interpretation of the foreign body. WY: acquired the data and obtained informed written consent from the patient for the publication of the clinical image.

## Data Availability

The data sharing not applicable to this article as no datasets were generated or analyzed during the current study.
